# Intraindividual comparison of circumferential strain using speckle tracking by echocardiography versus CMR feature tracking and myocardial tagging in patients

**DOI:** 10.1186/1532-429X-17-S1-P340

**Published:** 2015-02-03

**Authors:** Christopher Schneeweis, Adelina Doltra, Sarah B Nasser, Jan-Hendrik Hassel, Michael Gräfe, Ernst Wellnhofer, Bernhard Schnackenburg, Alexander Berger, Rolf Gebker, Eckart Fleck, Sebastian Kelle

**Affiliations:** 1 Cardiology, German Heart Institute Berlin, Berlin, Germany; 2 Cardiology, Dar Al Fouad Hospital, Cairo, Egypt; 3 Philips Health Care, Hamburg, Germany

## Background

Left ventricular myocardial motion analysis and the assessment of regional dysfunction are crucial for the detection and prognosis in different cardiac pathologies. Myocardial tagging (MT) is a cardiac magnetic resonance (CMR) based method for tracking myocardial motion, which has been established as reference standard for assessment of strain. Currently, strain can also be assessed by CMR using feature tracking (FT) or by echocardiography with speckle tracking (ST). Aim of our study was to evaluate intraindividually and compare myocardial circumferential strain (Ecc) using MT, FT and ST.

## Methods

In total 74 CMR examinations of 69 patients were included (25 female; 44.4 years average age), 45 patients had diagnosed phenylketonuria (PKU), the remaining 24 patients had arterial hypertension and were considered for sympathetic nerve modulation. All patients underwent a standardized CMR (1.5 T Philips Achieva) and transthoracic echocardiography (TTE, Philips IE3, offline analysis with QLAB). The circumferential strain (Ecc) was assessed with MT, FT and ST from the mid short axis (SAX). A global strain and segmental based analysis was performed. Segments with poor image quality were excluded (for MT 2, for FT 9 and for ST 56); for global strain and strain rate analysis only data from patients in which all segments were assessable were used. Bland Altman analyses were used for comparison of the three different techniques.

## Results

**Figure 1:**
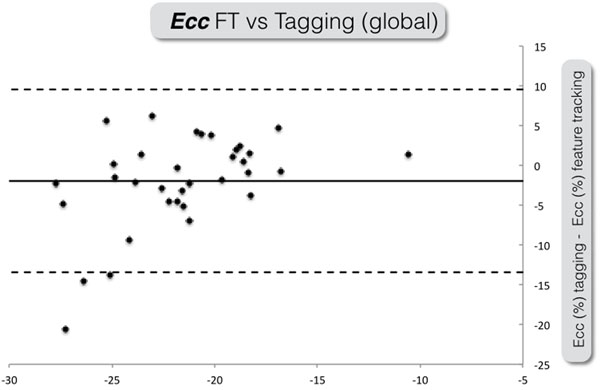
Bland Altman plot for global Ecc.

**Figure 2:**
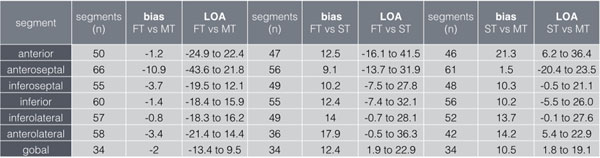


In 34 patients (204 segments) global strain values for all techniques were available (MT: -20.6 ± 3.4%, FT: -22.6 ± 5.6%, ST -10.1 ± 2.9%) and showed a moderate agreement (bias -2, LOA -13.4 to 9.5) between MT and FT (Figure 1), while no reasonable agreement was observed between FT and ST (bias 12.4, LOA 1.9 to 22.9) and MT and ST (bias 10.4, LOA 1.8 to 19.1). The segment based analysis (Figure 2) revealed a poor agreement between FT and tagging for all segments, while no agreement was observed for ST and FT, as well as for ST and FT. While there was no agreement between CMR based techniques and ST, only strain rates assessed by FT and MT were compared (bias 0.5, LAO -1.3 to 0.5) and showed a moderate agreement.

## Conclusions

Ecc values assessed by CMR based techniques (FT and MT) showed a moderate agreement for global Ecc, while a poor agreement was observed for the segmental analysis. No agreement, neither on global nor segmental level, was observed between CMR based techniques and ST. In summary the results of the three different techniques are not comparable.

## Funding

No funding.

